# Effect of Fireweed (*Chamerion angustifolium* L.) Extracts and Oenothein B on Colon Cancer Cells: Impact of Leaf Fermentation on Viability and Mitochondrial Function

**DOI:** 10.3390/medicina61111957

**Published:** 2025-10-31

**Authors:** Dovile Uogintaite, Rasa Baniene, Aldona Jasukaitiene, Mindaugas Marksa, Marius Lasinskas, Elvyra Jariene, Sonata Trumbeckaite

**Affiliations:** 1 Laboratory of Biochemistry, Neuroscience Institute, Lithuanian University of Health Sciences, Sukileliu Av. 13, LT-50162 Kaunas, Lithuania; 2 Department of Biochemistry, Medical Academy, Lithuanian University of Health Sciences, Eiveniu St. 4, LT-50161 Kaunas, Lithuania; 3 Surgical Gastroenterology Laboratory, Institute of Digestive Research, Lithuanian University of Health Sciences, Eiveniu St. 4, LT-50161 Kaunas, Lithuania; 4 Department of Analytical and Toxicological Chemistry, Medical Academy, Lithuanian University of Health Sciences, Sukileliu St. 13, LT-50166 Kaunas, Lithuania; 5 Department of Plant Biology and Food Sciences, Agriculture Academy, Vytautas Magnus University, Donelaicio St. 58, LT-44248 Kaunas, Lithuania; 6 Bioeconomy Research Institute, Agriculture Academy, Vytautas Magnus University, Donelaicio St. 58, LT-44248 Kaunas, Lithuania; 7 Department of Pharmacognosy, Medical Academy, Lithuanian University of Health Sciences, Sukileliu St. 13, LT-50166 Kaunas, Lithuania

**Keywords:** fireweed, colon cancer, mitochondria, viability, fermentation, oenothein B

## Abstract

*Background and Objectives:* Fireweed (*Chamerion angustifolium* L.) possesses antibacterial, antioxidant, anti-inflammatory, and anti-cancer properties. This study evaluated the effects of aqueous fireweed leaf extracts and their major compound, oenothein B, on the viability and mitochondrial function of Caco-2 colon cancer cells, emphasizing the impact of leaf fermentation. *Materials and Methods*: Cells were treated for 48 h with oenothein B and aqueous extracts from unfermented (NF) and fermented leaves (F 24 h, F 48 h). Cell viability and mitochondrial function were assessed by MTT assay and high–resolution respirometry. *Results*: IC_50_ values were 0.843 mg/mL (NF), 1.548 mg/mL (F 24 h), 1.931 mg/mL (F 48 h), and 0.09 mg/mL (57 µM) (oenothein B). Mitochondrial respiration decreased in up to 67% (glutamate/malate) and 61% (succinate) in both fermented and unfermented groups, while oenothein B increased in leak respiration by 34–73% but reduced oxidative respiration by 24%. *Conclusions:* Aqueous extracts of fireweed from both unfermented and fermented leaves significantly reduced Caco-2 cell viability and mitochondrial function. Oenothein B on its own had a stronger effect on cell viability, but a weaker effect on mitochondrial function, compared to fireweed extracts.

## 1. Introduction

Colon cancer is the third most diagnosed cancer and the second leading cause of cancer-related deaths worldwide [1]. In 2022, approximately 1.9 million new cases and 0.9 million deaths were reported, accounting for 9.6% of all cancer cases and 9.3% of cancer deaths globally [1,2]. By 2040, new cases and deaths are projected to rise to 3.2 million and 1.6 million, respectively, reflecting increases of 63% and 73.4% compared to 2020 [1].

Colon cancer originates from the hyperproliferation of mucosal epithelial cells, forming polyps or adenomas; about 10% of these progress to cancer as they grow larger. Adenocarcinoma, the invasive form, constitutes 96% of colon cancer cases [3]. While surgical resection is the primary treatment, recurrence occurs in 30–40% of cases, with 40–50% of recurrences appearing within the first few years after surgery [4]. Radiotherapy and chemotherapy, though effective against cancer cells, also damage healthy tissues, and multidrug resistance further reduces treatment efficacy, often leading to failure [5]. These challenges underscore the urgent need for novel colon cancer treatment strategies. One promising direction involves the exploration of natural compounds that selectively target cancer cell metabolism, particularly mitochondrial pathways, which are often dysregulated in cancer cells.

**Figure 1:**
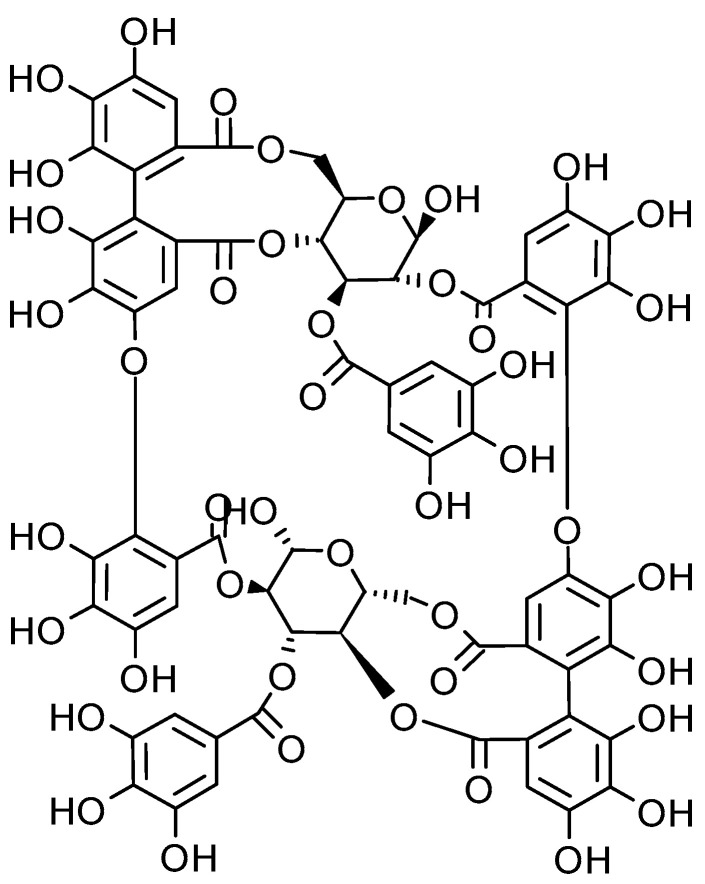
Chemical structure of oenothein B.

Fireweed (*Chamerion angustifolium* L.) is included in a European Medicines Agency (EMA) monograph stating that it can be used to relieve lower urinary tract symptoms associated with benign prostatic hyperplasia [6]. Fireweed contains abundant secondary metabolites, particularly polyphenolic compounds such as tannins, flavonoids, and phenolic acids. The diversity and abundance of these bioactives suggest that fireweed may exert pleiotropic effects at the cellular level, impacting several molecular pathways simultaneously. The tannin oenothein B (Figure 1) is believed to be the primary compound responsible for its anti-androgenic, anti-proliferative, anticancer, antioxidant, anti-inflammatory, and antibacterial properties [7,8]. Studies show that fireweed extracts exhibit anti-proliferative effects in both normal and cancer cells, including LNCaP (androgen-dependent), PZ-HPV-7 (androgen-independent), 1321N1 (astrocytoma), and HMEC (normal mammary cells) [9]. In LNCaP cells, fireweed extracts activated the apoptotic cascade by disrupting mitochondrial membranes [10]. Additionally, oenothein B has been shown to significantly reduce the growth of various cancer cell lines, such as KB (oral epidermoid), HeLa (cervical), DU-145 (prostate carcinoma), Hep-3B (hepatocellular), and HL-60 (promyelocytic leukemia), while exhibiting lower cytotoxicity to normal cells (WISH) [11]. These findings underscore fireweed’s potential as an anticancer agent, particularly for colon cancer, due to its ability to inhibit cancer cell growth with minimal impact on normal cells [10,11]. However, the precise mechanisms by which fireweed and its key constituents affect cellular targets, particularly mitochondria, remain insufficiently explored.

Despite these known benefits, little is understood about how fermentation—a process commonly used in traditional herbal medicine—modulates fireweed’s bioactive compounds and enhances its therapeutic effects. Fermentation, particularly solid-phase fermentation, is widely used in the food industry to modify plant properties such as smell, taste, and color and to enhance substance extraction [12]. This creates functional foods and beverages beneficial for health [13], which are mild in action and difficult to overdose [14]. Tea made from fermented fireweed leaves is an example of solid-phase fermentation improving its composition [15].

Solid-phase fermentation is carried out without adding water; instead, crushed and pressed leaves promote enzymatic degradation. Lasting 1–3 days, this process induces both intracellular biochemical changes and strong microbial activity. Enzymes produced by lactic acid bacteria, yeasts, and plant enzymes such as polyphenol oxidase break down macromolecules (proteins, lipids, polysaccharides) into low-molecular-weight compounds and secondary metabolites. Fermentation—whether aerobic or anaerobic—alters the phenolic profile by degrading complex molecules and enhancing compound bioavailability. In fireweed, solid-phase fermentation markedly affects polyphenol, flavonoid, and oenothein B content; anaerobic conditions increase these compounds the most, whereas aerobic fermentation yields an extract with the highest antioxidant activity [15].

All living cells, whether healthy or cancerous, require energy to function. This energy is primarily produced through glycolysis in the cytoplasm and/or oxidative phosphorylation in the mitochondria. Currently, there is a lot of research on fireweed’s effect on cancer cell proliferation; however, there is little data on its effect on cancer cell mitochondrial function. The development of natural-origin therapeutic agents that modulate the mitochondrial function of cancer cells as well as cancer cell viability is crucial, as it can open new avenues for more effective cancer treatments and improve patient survival outcomes. Alternatively, it could be used for cancer prevention, offering a proactive approach to reducing the risk of cancer development. There is a limited number of studies investigating the effects of fireweed extract on mitochondrial function in colon cancer cells. Despite the promising preliminary evidence, critical knowledge gaps persist regarding the specific effects of fireweed on mitochondrial energy metabolism in cancer cells, especially under different processing conditions, such as fermentation.

Given the increasing interest in the biological properties of fireweed and the potential of fermentation to boost bioactivity, this study aims to investigate the effects of unfermented and fermented fireweed leaf extracts, and their main component oenothein B, on the viability and mitochondrial function of Caco-2 colon cancer cells. Specifically, we seek to investigate whether fermentation alters the phytochemical composition of fireweed and its subsequent impact on cancer cell viability and mitochondrial function.

## 2. Materials and Methods

### 2.1. Chemicals

Ethylene glycol-bis-(b-aminoethylether)-*N*,*N*,*N*′*N*′-tetraacetic acid (EGTA), MgCl_2_, lactobionic acid, taurine, KH_2_PO_4_, Tris were obtained from “Sigma-Aldrich” (St. Louis, MO, USA). Sucrose and HEPES (4-(2-hydroxyethyl)-1-piperazineethanesulfonic acid) were obtained from “Carl Roth GmbH” (Karlsruhe, Germany).

Glutamic acid, malic acid, digitonin, adenosine-5‘-diphosphate sodium salt (ADP), succinic acid, cytochrome c from bovine heart, carboxytractyloside potassium salt, 2,4-dinitrophenol were obtained from “Sigma-Aldrich” (St. Louis, MO, USA).

Oenothein B was obtained from “Sigma-Aldrich” (St. Louis, MO, USA).

### 2.2. Plant Materials

The plant material was collected from Giedres Nacevicienes organic farm in 2023, located in Safarkos village, Jonava district, Lithuania. A section on a farm was left for fireweed to grow naturally by itself. The collected raw material was separated into the following 3 samples: unfermented, fermented for 24 h, and 48 h. The solid-phase fermentation was performed under aerobic conditions.

### 2.3. Solid-Phase Fermentation Process

Using specialized plastic blades, fresh fireweed leaves were chopped for the solid-phase fermentation process. The resultant raw material was then split into three samples. The fermented ready bulk was firmly packed into glass jars and sealed with an air-passing lid. For 24 and 48 h, the fermentation process was conducted in the chamber at 30 °C. After fermentation, the raw materials were lyophilized in a ZIRBUS sublimation dryer 3 × 4 × 5 (ZIRBUS Technology, Bad Grund, Germany) and frozen at −35 °C. For further analysis, the lyophilized leaves were ground into a powder using a Grindomix GM 200 laboratory mill (Retsch GmbH, Haan, Germany). The plant material powder was provided by the VMU Agriculture Academy, Department of Plant Biology and Food Sciences, scientific group.

### 2.4. Caco-2 Cell Line and Growing Conditions

Colorectal adenocarcinoma epithelial cell line Caco-2 was obtained from American Type Cell Culture (ATCC, Manassas, VA, USA). The cells were maintained in Ham’s F-12 medium (Gibco, Life Technologies Limited, Paisley, UK) with 10% fetal bovine serum (Gibco Life Technologies Limited, Paisley, UK) and 1% penicillin/streptomycin solution (Gibco Life Technologies Limited, Paisley, UK). Cells were cultivated in monolayers in sterile flasks/plates in an incubator at 37 °C in 5% CO_2_ humidity.

### 2.5. Fireweed Extract Preparation

Aqueous fireweed extracts were prepared from the provided plant material powder and non-sterile F-12 cell medium (concentration 25 mg/mL). The prepared solution was left at room temperature for 1 h. Afterwards, the solution was centrifuged, and the obtained supernatant was filtered using a 0.22 µm filter. The obtained extract was diluted to reach the required concentrations for cell treatment.

### 2.6. Oenothein B Preparation

A stock solution of oenothein B was prepared with non-sterile F-12 cell medium (concentration 1 mg/mL or 637 µM). The obtained solution was filtered using a 0.22 µm filter. The stock solution was diluted to reach the required concentrations for cell treatment.

### 2.7. Fireweed Extract Phytochemical Analysis Using HPLC

The identification and quantitative analysis of the active compounds were performed by high-performance liquid chromatography (HPLC). A Waters Alliance system, a 2695 chromatographic system with a 2998 diode-array detector, and an ACE 5 C18 chromatography column (250 × 4.6 mm) were used. Data was processed by Empower 3 Chromatography Data Software (version 3.9.0) (Waters Corporation, Milford, MA, USA). The eluent system consists of 100% acetonitrile and 0.1% trifluoroacetic acid, injection volume 10 µL, column temperature 25 °C, mobile phase flow rate 1 mL/min. The active compounds in the sample were identified by the retention time of the analytes and spectral data in the working range 200–400 nm.

### 2.8. Fireweed Extract and Oenothein B Treatment of Cells and IC_50_ Measurement

Cells were seeded in 96-well cell culture plates (1.2 × 10^4^ cells/well) and incubated for 24 h, maintaining the above-described conditions. After incubation, the cells were treated with varying concentrations of aqueous fireweed extract (0.5–3 mg/mL) and oenothein B (0.01–0.1 mg/mL) to test the half-maximal inhibitory concentration (IC_50_). A portion of the cancer cells remained untreated and were used as a control group. Cells were incubated for 48 h in the same conditions. MTT assay was used to determine IC_50_ for each fireweed extract. No less than 3 replicates of the experiment were carried out. The IC_50_ was calculated using Quest Graph IC_50_ Calculator (AAT Bioquest, Inc., Sunnyvale, CA, USA).

### 2.9. MTT Metabolic Activity Assay

Cell viability was assessed by MTT (3-(4,5-dimethylthiazol-2-yl)-2,5 diphenyltetrazolium bromide) (Invitrogen, Carlsbad, CA, USA) assay. After treatment, the old growth medium was removed and new medium with MTT was added (MTT concentration of 0.5 mg/mL). The cells were incubated for 3 h at 37 °C. After incubation, the medium with MTT was removed, and the remaining formazan crystals were dissolved in 100 µL DMSO (dimethyl sulfoxide) (Carl Roth GmbH, Karlsruhe, Germany) by agitation in the spectrophotometer for 90 s. The absorbance was measured with the spectrophotometer (The Sunrise (Software v7.1), Tecan, Grodig, Austria) at a wavelength of 570/620 nm. Colorimetric absorption values were compared to the untreated control group.

### 2.10. Measurement of Mitochondrial Function in Cancer Cells

**Figure 2:**
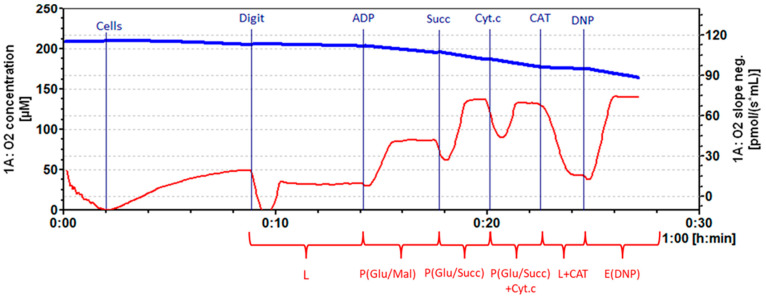
Typical mitochondrial respiration graph. The blue line indicates oxygen concentration (µM) in the medium; the red line indicates respiratory flux—oxygen consumption per unit of chamber volume (pmol/(s·mL)). L—mitochondrial respiration rate in leak state, after adding 1 mg/mL of Digitonin; P(Glu/Mal)—mitochondrial oxidative phosphorylation rate after adding 1 mM ADP, in the presence of Complex 1 substrates glutamate and malate; P(Glu/Succ)—mitochondrial oxidative phosphorylation rate after adding 15 mM Complex 2 substrate succinate; P(Glu/Succ) + Cyt.c—mitochondrial oxidative phosphorylation rate after adding 32 µM cytochrome c; L + CAT—mitochondrial respiration rate in leak state after adding 0.75 µM carboxyatractyloside (CAT); E(DNP)—mitochondrial respiration rate efficacy after adding 0.3 mM 2,4-dinitrophenol (DNP).

Caco-2 cells were treated with a determined IC_50_ of fireweed extracts and oenothein B for 48 h. A portion of the cancer cells was left untreated and used as a control group. Mitochondrial respiration (oxygen consumption) rate was recorded by high–resolution respirometry system Oxygraph-2k (OROBOROS Instruments, Innsbruck, Austria) at 37 °C in the MiR06 medium (0.5 mM EGTA, 3 mM MgCl_2_, 60 mM lactobionate, 20 mM Taurine, 10 mM KH_2_PO_4_, 20 mM HEPES, 110 mM sucrose (pH 7.1 at 30 °C)). Digitonin (16 µg/mL) was added and incubated for 5 min to permeabilize the cell membrane. Mitochondrial respiration rate in Leak state (L) was recorded in the medium with cells and mitochondrial Complex 1 substrate (5 mM glutamate + 2 mM malate). Maximal oxidative phosphorylation rate (P(Glu/Mal)) in the presence of Complex 1 substrates was determined by adding 1 mM ADP. Complex 2 substrate succinate (12 mM) was used to achieve maximal mitochondrial oxidative phosphorylation (P(Glu/Succ)) in the presence of Complex 1 and II substrates. The effect of cytochrome c on respiration rate (to identify mitochondrial outer membrane permeability) was determined by adding 32 μM cytochrome c (P(Glu/Succ) + Cyt.c). 0.75 µM of carboxyatractyloside (CAT) was added to assess the permeability of the inner mitochondrial membrane (L + CAT). To determine the respiration chain effectiveness, 0.3 mM of 2,4-dinitrophenol (DNP) was added to the medium (E(DNP)). The respiratory control index (RCI) for glutamate/malate was calculated as the ratio between P(Glu/Mal)/L respiration rate. RCI for succinate was calculated from the ratio P(Glu/Succ) + Cyt.c/L + CAT. Cytochrome c effect was calculated as the ratio P(Glu/Succ) + Cyt.c)/P(Glu/Succ). Datlab 7 software (version 7.4.0.4) (OROBOROS Instruments, Innsbruck, Austria) was used for real-time data acquisition and data analysis. Oxygen consumption was related to cell numbers (pmol/s/0.7 mLn cells) (Figure 2).

### 2.11. Statistical Analysis

Statistical analyses were performed using IBM SPSS Statistics 29.0.1.0 (IBM, Chicago, IL, USA). The data are presented as mean ± SD of three or more independent experiments. The level of statistical significance was set as *p* < 0.05.

## 3. Results

### 3.1. Phytochemical Analysis of Fireweed Aqueous Extract

The phytochemical content of aqueous fireweed extracts was obtained by performing HPLC analysis. Phytochemical analysis revealed significant differences in the concentrations of bioactive compounds between the unfermented and fermented extracts. Table 1 shows the detected compounds, including the ellagitannin oenothein B, phenolic acids such as neochlorogenic acid, chlorogenic acid, 4-*O*-caffeoylquinic acid, caffeic acid, ellagic acid, and coumaric acid, as well as the flavonoids hyperoside and isoquercitrin. The concentrations of the detected compounds varied and decreased depending on the duration of the fermentation. The aqueous extract from unfermented leaves had the highest concentration of oenothein B (568.42 µg/mL), which decreased by 65% and 83% after 24 and 48 h of fermentation. This decrease was also observed in phenolic acids, such as chlorogenic acid and neochlorogenic acid, the levels were reduced by 2.3-fold and 2.4-fold after 24 h of fermentation and by 3.4-fold and 3.7-fold after 48 h of fermentation, respectively, (Table 1). In contrast, the content of ellagic acid increased by 1.2-fold after 24 h and by 1.3-fold after 48 h of fermentation. The amounts of flavonoids, specifically hyperoside and isoquercitrin, were higher in the aqueous extract obtained from unfermented fireweed leaves, showing increases of 2.0-fold and 1.6-fold.

### 3.2. Determination of the Half-Maximal Inhibitory Concentration (IC_50_) of Fireweed Extract in Caco-2 Cells

**Figure 3:**
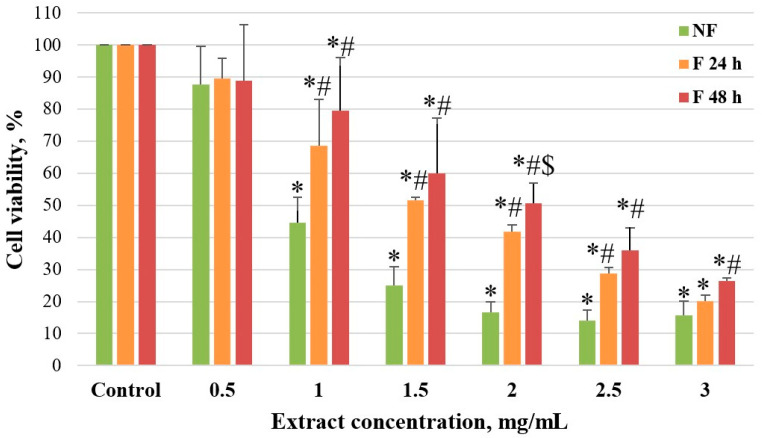
Effect of fireweed leaf aqueous extract on Caco-2 cell viability. Cell viability analysis by MTT assay. Cell viability (%) depending on aqueous fireweed extract concentration (mg/mL) after 48 h treatment. NF—cells treated with unfermented fireweed leaf extract; F 24 h—cells treated with extract made from fireweed leaves fermented for 24 h; F 48 h—cells treated with extract made from fireweed leaves fermented for 48 h. *n* ≥ 3, MEAN ± SD; * *p* < 0.05 compared to control; # *p* < 0.05 compared to NF group; $ *p* < 0.05 compared to F 24 h group.

The metabolic activity of Caco-2 cells was determined by MTT assay. We found that both fermented and unfermented fireweed extracts inhibited cancer cell viability; however, the unfermented fireweed extract significantly reduced Caco-2 cell viability compared to the fermented extracts. Cell viability showed an approximate 12% reduction at a 0.5 mg/mL extract concentration across all groups. Although this decrease was not statistically significant, it exhibited a clear tendency toward reduced viability. An increase in the extract concentration to 1 mg/mL led to a significant reduction in cell viability, with pronounced differences observed between the groups. In the NF group, cell viability decreased by 55%, in the F 24 h group by 31%, and in the F 48 h group by 20%. This trend continued with higher extract concentrations, with NF showing the strongest effect and F 48 h—the weakest. At the highest concentration of 3 mg/mL, the effect on cell viability became more comparable across groups. The NF group showed the greatest reduction in cell viability (up to 84%), while the F 24 h and F 48 h groups showed lesser reductions of 80% and 74%. Thus, the IC_50_ for unfermented extract was 0.843 mg/mL, while fermentation for 24 h and 48 h increased the IC_50_ to 1.548 mg/mL and 1.931 mg/mL, respectively, (Figure 3). This suggests that fermentation reduces the cytotoxic potency of the extract, potentially due to a decrease in the concentration of oenothein B and other polyphenols that may exhibit anticancer activity.

### 3.3. Determination of the Half-Maximal Inhibitory Concentration (IC_50_) of Oenothein B in Caco-2 Cells

**Figure 4:**
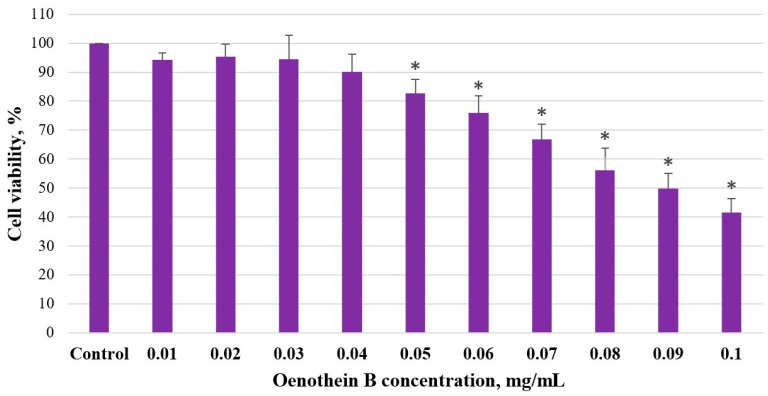
Effect of oenothein B on Caco-2 cell viability. Cell viability analysis by MTT assay. Cell viability (%) depending on oenothein B concentration (mg/mL) after 48 h treatment. *n =* 4, MEAN ± SD; * *p* < 0.05 compared to control.

The metabolic activity of Caco-2 cells treated with oenothein B was determined by the MTT assay. We detected that oenothein B also inhibited Caco-2 cell viability—it decreased in statistical significance by 17% at a 0.05 mg/mL oenothein B concentration. By increasing oenothein B concentration, the Caco-2 cell viability continued to decrease in showing that the effect is dose-dependent. At the highest concentration of 0.1 mg/mL, the cell viability was reduced by 58%, *p* < 0.05. It was calculated that IC_50_ for oenothein B was 0.09 mg/mL (57 µM) (Figure 4).

### 3.4. Effects of Fireweed Leaf Extract and Oenothein B on Mitochondrial Function in Caco-2 Cells

The IC_50_ values of aqueous fireweed extracts, derived from unfermented leaves (NF) and leaves fermented for 24 h (F 24 h) and 48 h (F 48 h) under aerobic conditions, were determined to be 0.843 mg/mL, 1.548 mg/mL, and 1.931 mg/mL. The IC_50_ value of oenothein B was 0.09 mg/mL (57 µM). These IC_50_ concentrations were then applied in experiments to assess their effects on mitochondrial function in Caco-2 cells. The mitochondrial respiration rate was assessed in the leak (L) state and during oxidative phosphorylation (P) using Complex 1 (glutamate/malate) and Complex 2 (succinate) substrates. The permeability of the outer and inner mitochondrial membranes was evaluated by the addition of cytochrome c and CAT (carboxyatractyloside). The efficacy of the mitochondrial respiratory chain was analyzed using DNP (2,4-dinitrophenol). Detailed protocols for these measurements are provided in Figure 2 of the Section 2.

**Figure 5:**
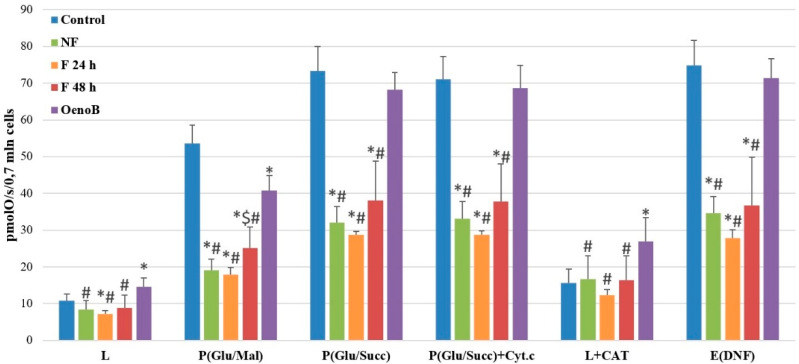
Fireweed leaf aqueous extract and oenothein B effect on Caco-2 cell mitochondrial function. NF—cells treated with unfermented fireweed leaf extract; F 24 h—cells treated with extract made from fireweed leaves fermented for 24 h; F 48 h—cells treated with extract made from fireweed leaves fermented for 48 h; OenoB—cells treated with oenothein B. L—proton leak state with mitochondrial Complex 1 substrates (glutamate/malate); P(Glu/Mal)—oxidative phosphorylation with mitochondrial Complex 1 substrates (glutamate/malate) after adding ADP; P(Glu/Succ)—oxidative phosphorylation with mitochondrial Complex I (glutamate/malate) and Complex 2 (succinate) substrates; P(Glu/Succ + Cyt.c)—oxidative phosphorylation with cytochrome c; L + CAT—proton leak state with the inhibitor adenine nucleotide carrier carboxyatractyloside (CAT); E(DNP)—electron transport state with the uncoupler dinitrophenol (DNP). *n* ≥ 3, MEAN ± SD; * *p* < 0.05 compared to control; $ *p* < 0.05 compared to F 24 h group; # *p* < 0.05 compared to oenothein B.

High–resolution respirometry revealed that fireweed extracts significantly inhibited mitochondrial function in Caco-2 cells. Fireweed extract from fermented leaves (group F 24 h) caused a statistically significant decrease in respiration rate in leak state (L) by 34%, compared to the control group (*p* < 0.05). A decrease in respiration rate in this state was also noticed in other groups (NF and F 48 h); however, it was not statistically significant. The oxidative phosphorylation rate with glutamate/malate as substrate (P(Glu/Mal)) was significantly decreased in all treated groups. The respiration rate decreased by 64% in the NF group, 67% in the F 24 h group, and 53% in the F 48 h group (*p* < 0.05). A similar effect was determined in the oxidative phosphorylation rate with succinate/glutamate/malate as substrate (P(Glu/Succ)). Mitochondrial respiration rate was reduced by 56% in the NF group, 61% in the F 24 h group, and 48% in the F 48 h group (*p* < 0.05) (Figure 5).

Similarly, oenothein B had a significant effect on mitochondrial respiration in Caco-2 cells. Specifically, an increase in the respiration rate in the leak state (L) of 34% compared to the control group (*p* < 0.05), i.e., without pre-treatment. Moreover, the oxidative phosphorylation rate with Complex 1 substrates glutamate/malate (P(Glu/Mal)) was significantly reduced by 24% (*p* < 0.05). In contrast, respiration with succinate (P(Glu/Succ)) and cytochrome c (P(Glu/Succ)+ Cyt.c) did not show any statistically significant changes in the onethein B-treated group and remained comparable to the control.

**Figure 6:**
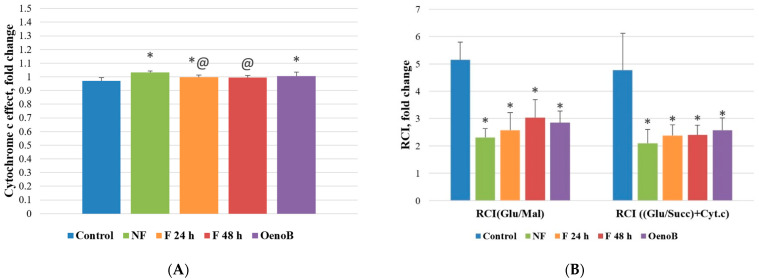
Fireweed leaf aqueous extract and oenothein B effect on Caco-2 cell cytochrome c effect (**A**) and respiratory control index (**B**). NF—cells treated with unfermented fireweed leaf extract; F 24 h—cells treated with extract made from fireweed leaves fermented for 24 h; F 48 h—cells treated with extract made from fireweed leaves fermented for 48 h; OenoB—cells treated with oenothein B. RCI(Glu/Mal)—respiratory control index in the presence of Complex 1 substrates (glutamate/malate); RCI((Glu/Succ) + Cyt.c))—respiratory control index in the presence of Complex 2 substrate (succinate). *n* = 3, MEAN ± SD; * *p* < 0.05 compared to control; @ *p* < 0.05 compared to NF.

It is interesting to note that the respiration rate after adding cytochrome c (P(Glu/Succ) + Cyt.c) remained practically the same after pretreatment with fireweed extract, and there was only a slight increase in cytochrome c effect (by 6%, *p* < 0.05) in NF group and (by 3%, *p* < 0.05) in F 24 h group, but this effect was minimal. Oenethein B has a similar effect—there was a slight 4% increase in cytochrome c effect (*p* < 0.05) as compared to the control (untreated group) (Figure 6A). This could suggest just a slight increase in mitochondrial outer membrane permeability after pretreatment with fireweed extract and oenethein B.

Furthermore, there was no significant increase in the inner mitochondrial membrane permeability in fireweed extract-treated groups after adding carboxyatractyloside (CAT), an inhibitor of the ADP/ATP translocator (L + CAT), as this parameter remained unchanged. In contrast, the leak respiration rate in oenethein B-treated group was markedly elevated (by 73%), indicating an increase in the permeability of the mitochondrial inner membrane.

After the addition of the uncoupler 2,4-dinitrophenol (DNP), the respiration rate (E(DNP)) in the fireweed-treated groups decreased of 54%, 63%, and 51% (NF, F 24 h, and F 48 h groups, respectively), (*p* < 0.05), compared to the control, indicating damage to the mitochondrial respiratory chain. There was no significant change in respiration rate E(DNP) after pretreatment with oenethein B (Figure 5).

We found, that fireweed extracts caused the decrease in respiratory control index with glutamate/malate as substrate RCI(Glu/Mal)—it decreased from 5.15 ± 0.65 in the control group to 2.31 ± 0.32 (a 55% reduction) in the NF group, 2.57 ± 0.66 (a 50% reduction) in the F 24 h group, and 3.04 ± 0.66 (a 41% reduction) in the F 48 h group (Figure 6B). Similarly, the respiratory control index when oxidizing succinate RCI((Glu/Succ) + Cyt.c) was reduced by 56% in the NF group (2.10 ± 0.50), 50% in the F 24 h group (2.37 ± 0.39), and 50% in the F 48 h group (2.40 ± 0.36), compared to the control group (4.77 ± 1.35). After treatment with oenothein B the respiratory control index RCI(Glu/Mal) was reduced by 45% (2.85 ± 0.43), while RCI((Glu/Succ) + Cyt.c)—by 46% (2.57 ± 0.45) (*p* < 0.05) (Figure 6B), showing the damage of mitochondrial function in both, fireweed treated and oenethein B treated groups.

## 4. Discussion

Interest in fireweed as a medicinal plant has increased in its pharmacological properties and potential anticancer effects. Studies show that fireweed extract reduces cancer cell proliferation with minimal effects on healthy cells [10,11]. Since mitochondria are central to cancer cell metabolism, understanding their response to fireweed extract is crucial, though data on its impact is limited. Moreover, little research exists on how fermentation affects the biological properties of fireweed extract, despite its common use in traditional medicine. This study aimed to evaluate the effects of fireweed extract and its main component, oenothein B, on Caco-2 colon cancer cell viability and mitochondrial function, and assess whether fermentation alters these effects. The increasing burden of treatment resistance and relapses in colon cancer therapy has prompted a search for novel agents that selectively target cancer cell metabolism without harming healthy cells.

Our study showed that both unfermented and fermented fireweed leaf extracts inhibited Caco-2 colon cancer cell viability in a concentration-dependent manner, with the strongest effect observed for the unfermented extract (84% reduction at 3 mg/mL; IC_50_ 0.843 mg/mL). The greater cytotoxicity of the unfermented extract may be related to its higher polyphenol and oenothein B content. Oenothein B alone also reduced cell viability (58% at 0.1 mg/mL; IC_50_ = 0.09 mg/mL (57 µM)), but its concentration in the unfermented extract (≈0.02 mg/mL) was too low to explain the full effect. This suggests that other phytochemicals in the extract may act synergistically, contributing to its stronger overall anticancer activity.

M. Perużyńska et al. [16] evaluated the antiproliferative effects of ethanolic fireweed extract on normal human fibroblasts (HDF) and various cancer cell lines as follows: melanoma (A375), breast (MCF7), colon (HT-29), lung (A549), and liver (HepG2). HT-29 cells were three times more sensitive than HDF and five times more sensitive than HepG2 and A549, suggesting selectivity for specific cancer types.

M. Stolarczyk et al. [17] showed that aqueous fireweed extracts strongly inhibited prostate cancer (LNCaP) cell growth (IC_50_ ≈ 35 µg/mL), supporting its traditional urogenital use. Kowalik et al. [18] found that digested extracts reduced HT-29 cell proliferation to 27% at 250 µg/mL after 96 h, while stimulating normal CCD 841 CoTr cells by 128%. Another study [19] reported that unfermented extract (3 mg/mL) decreased Caco-2 cell viability by 91%, and fermented extract by 75% (IC_50_ 0.81 mg/mL and 1.18 mg/mL, respectively). Our results are consistent with these findings but extend them by using aerobic fermentation for 24–48 h, instead of 72 h anaerobic conditions, thus reflecting more traditional, food-compatible processing.

Mitochondrial dysfunction plays a crucial role in cancer metabolism, and the observed disruption of oxidative phosphorylation indicates that fireweed extracts interfere with energy production essential for cancer cell survival. Inhibition of Complex 1 and 2-driven respiration led to reduced ATP synthesis and mitochondrial stress, promoting cell death. Fireweed extracts from both unfermented and fermented leaves markedly decreased in mitochondrial respiration (up to 67% with glutamate/malate and 61% with succinate), as well as oxidative phosphorylation and respiratory control indexes (by 41–56%), indicating impaired mitochondrial efficiency. Uncoupled respiration also declined by 51–63% (*p* < 0.05). Although minor mitochondrial membrane damage was observed, only small but significant changes in cytochrome c release were detected (6% in NF and 3% in F 24 h), suggesting slight outer membrane permeability differences between extracts.

Our study demonstrated that oenothein B, a key compound in fireweed, altered mitochondrial function mainly by increasing inner mitochondrial membrane permeability. It elevated leak respiration by 34% (L) and 73% (L + CAT) while moderately reducing oxidative phosphorylation by 24% and lowering the respiratory control index by ~45% (*p* < 0.05). Compared with oenothein B, fireweed extracts significantly reduced mitochondrial respiration (by 39–61%), oxidative phosphorylation (by 38–58%), and uncoupled respiration (by 49–61%), indicating a stronger inhibitory effect on mitochondrial activity. In contrast, oenothein B caused greater inner membrane damage, as reflected by higher leak respiration rates. No significant differences were observed in cytochrome c effect or respiratory control index between the treatments.

The fact that practically no statistically significant differences were observed between the fermented and unfermented groups indicates that aqueous fireweed extract effectively suppresses mitochondrial functions in Caco-2 cells, regardless of whether it is derived from fermented or unfermented leaves. The minimal difference in the effect between unfermented and fermented groups suggests that fermentation did not substantially alter the extract’s impact on mitochondrial function.

Comparisons between fireweed-treated groups and oenothein B-treated groups reveal that oenothein B on its own has a significantly weaker effect. This indicates that the phytochemical compounds detected in fireweed extract may have a synergistic effect that strengthens the inhibition of Caco-2 cell mitochondrial function.

In a previous study [19], researchers examined the mitochondrial response of Caco-2 colon cancer cells to aqueous fireweed leaf extract in two forms, both unfermented and fermented, for 72 h under anaerobic conditions. Oxidative respiration rate P(Glu/Mal) decreased by 52% (unfermented) and 40% (fermented), while P(Glu/Succ) was reduced by 35% and 27%. An increase in leak respiration rate (L + CAT) indicated inner mitochondrial membrane damage, with a slight increase in cytochrome c effect in the unfermented group, suggesting outer mitochondrial membrane damage. Both extracts similarly reduced respiratory control indexes (RCI) by 52–56% with glutamate/malate and succinate as substrates. Our findings show a slightly greater decrease in oxidative phosphorylation and RCI, but the trend remains consistent; unfermented extract exerts a stronger effect than fermented. This is the only study available in comparison with our results. Based on these findings, further investigation into the effects of fireweed extract on cancer cell mitochondrial function is necessary, as it has the potential to become a promising treatment option for colon cancer.

M. Lasinskas et al. [20] analyzed the phytochemical composition of fireweed collected from the same source and prepared under identical conditions as in our study. The methanolic extract of fireweed leaves analyzed by HPLC showed a 21% and 34% reduction in total polyphenols after 24 h and 48 h of aerobic fermentation. Oenothein B content slightly increased after 24 h but returned to baseline after 48 h, while phenolic acids decreased up to 47%, and flavonoids rose modestly (2–12%). In aqueous fireweed extracts investigated by us, oenothein B decreased more markedly—by 65% after 24 h and 83% after 48 h—correlating with the reduced cytotoxic effect on Caco-2 cells, as the unfermented extract with the highest oenothein B level showed the strongest antiproliferative activity.

Galambosi et al. [21] studied the impact of fermentation on compounds detected in fireweed. Fireweed leaves collected in 2012 and 2013 were aerobically fermented for ~44 h at 28 °C, 35 °C, and 40°. Total tannins decreased by 31–44%, flavonoids by 14–24%, hyperoside by 51–68%, and oenothein B by 50–71%. These findings highlight the need for further research into the effects of fermentation on fireweed’s bioactive profiles and its therapeutic potential in cancer treatment.

Overall, our results suggest that aqueous fireweed extract disrupts mitochondrial function and reduces colon cancer (Caco-2) cell viability through the combined actions of multiple phytochemicals. The fermentation process alters this composition but does not fundamentally change the extract’s effect on mitochondrial activity. Comparisons with oenothein B alone suggest only a partial effect on Caco-2 cell viability and mitochondrial function, supporting the hypothesis of a synergistic interaction among the constituents of fireweed extract.

The next objective is to investigate the detailed mechanism of action of the non-fermented fireweed leaf extract on Caco-2 cancer cells and to assess the potential synergistic interactions among its active compounds, as this represents a promising natural approach with anticancer potential.

## 5. Conclusions

In conclusion, aqueous fireweed leaf extracts, both fermented and unfermented, significantly reduced the viability and mitochondrial function of Caco-2 colon cancer cells by inhibiting Complex 1 and 2 respiration. Oenothein B, the main component of fireweed, also decreased cell viability and mitochondrial activity, but causes stronger inner membrane permeability changes and weaker oxidative phosphorylation inhibition than the whole extract. The stronger effect of the complete extract suggests a synergistic interaction among its bioactive compounds. Further research is needed to clarify the molecular mechanisms and therapeutic potential of fireweed extract in cancer.

## Figures and Tables

**Table 1:** The average phenolic compound contents (µg/mL) in aqueous fireweed leaf extracts based on different leaf fermentation time (h).

Fermentation	Oenothein B	Neochlorogenic Acid	Chlorogenic Acid	4-*O*-Caffeoylquinic Acid	Caffeic Acid	Ellagic Acid	Hyperoside	Isoquercitrin	Coumaric Acid
Unfermented	568.42	39.47	44.29	12.87	1.22	6.47	12.42	129.27	-
24 h fermentation	196.77	16.20	19.46	6.04	0.40	7.74	8.61	101.79	0.59
48 h fermentation	97.58	10.54	12.91	4.64	0.29	8.60	6.10	83.14	-

## Data Availability

The original contributions presented in this study are included in the article. Further inquiries can be directed at the corresponding author.

## Abbreviations

The following abbreviations are used in this manuscript:

ADP	adenosine diphosphate
ATP	adenosine triphosphate
CAT	carboxyatractyloside
D.M.	dry matter
DNP	2,4-dinitrophenol
E(DNP)	electron transport state with the uncoupler dinitrophenol (DNP)
EMA	European Medicines Agency
F 24 h	cells treated with extract made from fireweed leaves fermented for 24 h
F 48 h	cells treated with extract made from fireweed leaves fermented for 48 h
HPLC	high-performance liquid chromatography
IC_50_	half-maximal inhibitory concentration
L	proton leak state with mitochondrial Complex 1 substrates (glutamate/malate)
L + CAT	proton leak state with the inhibitor adenine nucleotide carrier carboxyatractyloside (CAT)
MTT	3-(4,5-dimethylthiazol-2-yl)-2,5 diphenyltetrazolium bromide
NF	cells treated with unfermented fireweed leaf extract
OenoB	cells treated with oenothein B
P(Glu/Mal)	oxidative phosphorylation with mitochondrial Complex 1 substrates (glutamate/malate) after adding ADP
P(Glu/Succ)	oxidative phosphorylation with mitochondrial Complex 1 (glutamate/malate) and Complex 2 (succinate) substrates
P(Glu/Succ + Cyt.c)	oxidative phosphorylation with cytochrome c
RCI((Glu/Succ) + Cyt.c)	respiratory control index when oxidizing succinate
RCI(Glu/Mal)	respiratory control index with glutamate/malate as substrate
RCI	respiratory control index
